# Epidrugs as Promising Tools to Eliminate *Plasmodium falciparum* Artemisinin-Resistant and Quiescent Parasites

**DOI:** 10.3390/pharmaceutics15102440

**Published:** 2023-10-10

**Authors:** Thibaud Reyser, Lucie Paloque, Michel Nguyen, Jean-Michel Augereau, Matthew John Fuchter, Marie Lopez, Paola B. Arimondo, Storm Hassell-Hart, John Spencer, Luisa Di Stefano, Françoise Benoit-Vical

**Affiliations:** 1 LCC-CNRS, Laboratoire de Chimie de Coordination, Université de Toulouse, CNRS, 31077 Toulouse, France; lucie.paloque@lcc-toulouse.fr (L.P.); reyser.thibaud@gmail.com (T.R.); michel.nguyen@lcc-toulouse.fr (M.N.); jean-michel.augereau@lcc-toulouse.fr (J.-M.A.); 2 MAAP, New Antimalarial Molecules and Pharmacological Approaches, Inserm ERL 1289, 31077 Toulouse, France; 3 Institut de Pharmacologie et de Biologie Structurale (IPBS), Université de Toulouse, CNRS, Université Toulouse III—Paul Sabatier (UPS), 31077 Toulouse, France; 4 Department of Chemistry, Molecular Sciences Research Hub, Imperial College London, White City Campus, London W12 0BZ, UK; m.fuchter@imperial.ac.uk; 5 Institut des Biomolécules Max Mousseron (IBMM), CNRS, Université de Montpellier, ENSCM UMR 5247, 34293 Montpellier, France; marie.lopez@cnrs.fr; 6 Epigenetic Chemical Biology, Department of Structural Biology and Chemistry, Institut Pasteur, Université de Paris-Cité, UMR 3523 CNRS, 75015 Paris, France; paola.arimondo@cnrs.fr; 7 Department of Chemistry, School of Life Sciences, University of Sussex, Falmer BN1 9QJ, UK; s.hassell-hart@sussex.ac.uk (S.H.-H.); j.spencer@sussex.ac.uk (J.S.); 8 MCD, Centre de Biologie Intégrative (CBI), Université de Toulouse, CNRS, UPS, 31062 Toulouse, France

**Keywords:** malaria, epigenetics, artemisinin resistance, quiescent parasites, epidrugs

## Abstract

The use of artemisinin and its derivatives has helped reduce the burden of malaria caused by *Plasmodium falciparum.* However, artemisinin-resistant parasites are able, in the presence of artemisinins, to stop their cell cycles. This quiescent state can alter the activity of artemisinin partner drugs leading to a secondary drug resistance and thus threatens malaria eradication strategies. Drugs targeting epigenetic mechanisms (namely epidrugs) are emerging as potential antimalarial drugs. Here, we set out to evaluate a selection of various epidrugs for their activity against quiescent parasites, to explore the possibility of using these compounds to counter artemisinin resistance. The 32 chosen epidrugs were first screened for their antiplasmodial activity and selectivity. We then demonstrated, thanks to the specific Quiescent-stage Survival Assay, that four epidrugs targeting both histone methylation or deacetylation as well as DNA methylation decrease the ability of artemisinin-resistant parasites to recover after artemisinin exposure. In the quest for novel antiplasmodial drugs with new modes of action, these results reinforce the therapeutic potential of epidrugs as antiplasmodial drugs especially in the context of artemisinin resistance.

## 1. Introduction

Last year, malaria claimed more than 600,000 lives according to the latest WHO Malaria report, and despite renewed efforts towards malaria elimination, the number of worldwide cases is growing [1]. Even though artemisinins (artemisinin and its derivatives, ART) have tremendously helped in reducing the malaria death toll, their broad use contributed to the emergence of *Plasmodium falciparum* resistance in South-East Asia [2]. Cases of resistance to both ART and their partner drugs are now common in Thailand and Cambodia [1,3]. Worryingly, reports in Africa state that cases of ART resistance have already appeared in several countries [1,4,5], thus fueling the fear of a wide-scale emergency and spread of ART resistance on the continent where the disease claims the majority of its victims. In contrast with usual mechanisms of drug resistance (often based on altering the drug transporter or target) [6], ART resistance involves highly complex and multifactorial mechanisms not yet fully understood [7,8]. This resistance is associated with mutations in the *pfk13* gene [9], and also relies on different mechanisms such as reduced uptake of hemoglobin [10], modification of the redox balance [11], activation of the unfolded protein response [12] leading to PK4 (*Plasmodium* kinase 4)-mediated phosphorylation of the eukaryotic transcription factor eIF2α [13], adjustment of the central carbon-linked pyruvate and glutamate metabolism in the mitochondrion [14], an increase in the amount of PfPI3K (phosphatidylinositol-3-kinase) leading to increased production of PI3P (phosphatidylinositol-3-phosphate) [15,16], and is associated with the up-regulation of PfCRK1 (a cyclin dependent kinase) [17]. Moreover, ART resistance seems to be associated with an extended ring stage of the parasites [12,18], which have a higher ability to survive ART exposure when they are younger rings [2,19,20,21]. All of these mechanisms finally allow a part of the parasite population to escape from ART action through a temporary growth-arrest and then to recover once ART has been eliminated. This cell cycle arrest, also called quiescence or dormancy, which involves only a sub-population and is reminiscent of persistence [22], is associated with a modified parasite metabolism activity leading to the unavailability of drug targets [14,23,24,25,26,27]. Indeed, we previously demonstrated that antiplasmodial drugs can be active against ART-resistant parasites at the proliferative state but not in a quiescent state [28]. Several key steps of the parasite intra-erythrocytic cell cycle depend on a fine epigenetic regulation of “just in time” gene expression [29]. Therefore, epigenetic effectors such as histone deacetylases (HDACs) or DNA methyltransferases (DNMTs) are considered as potential targets for new classes of antimalarials [30,31,32,33]. Considering the impact of ART on the course of the parasite cell cycle and the importance of epigenetics in its regulation, we set out to evaluate the antiplasmodial activity of a selection of epidrugs (i.e., drugs targeting epigenetic mechanisms) against ART-resistant parasites in both proliferative and quiescent states and thus the possibility of using them to counter artemisinin resistance. In the current context of ART-resistance spreading in malaria areas, finding new antiplasmodial drugs that are able to kill ART-resistant parasites, regardless of their metabolic activity, can be useful for malaria eradication.

**Figure 1:**
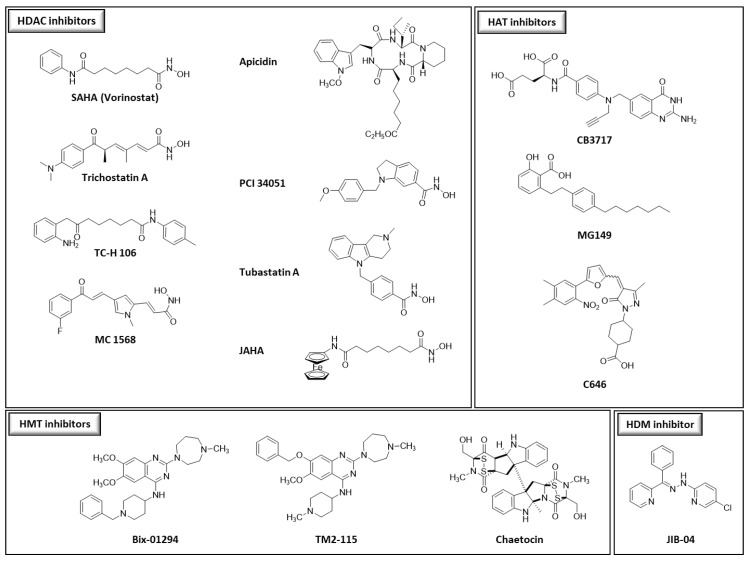

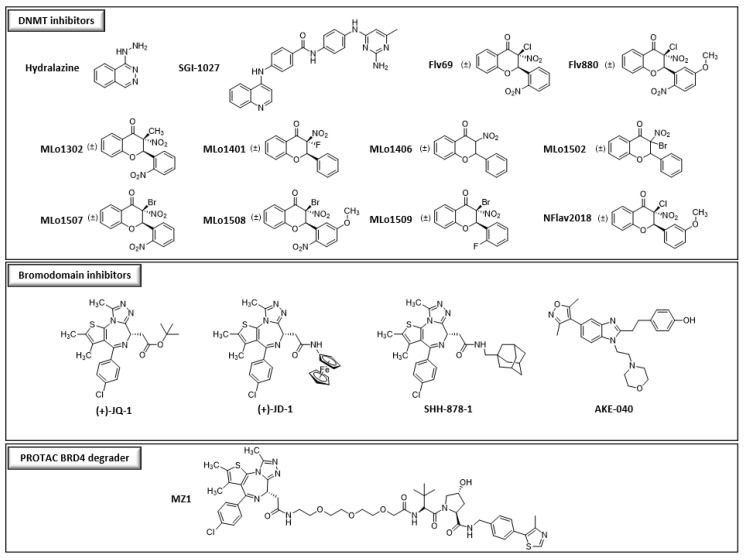
Structures of the different compounds tested in this study (HDACs: histone deacetylases; HMTs: histone methyltransferases; DNMTs: DNA methyltransferases; HATs: histone acetyltransferases; HDMs: histone demethylases, BRD4: bromodomain-containing protein 4).

We chose thirty-two drugs known or designed to target major enzymes and proteins involved in epigenetic regulation: eight inhibitors of histone deacetylases (HDACs), three of histone methyltransferases (HMTs), three of histone acetyltransferases (HATs), one of histone demethylase (HDM), twelve of DNA methyltransferases (DNMTs), four of bromodomains (reader proteins that recognize the acetylation lysine and participate with the activation of gene transcription) and one degrader of bromodomains (BRDs) (Figure 1). The choice of compounds was guided by their accessibility through commercial sources or through their design and synthesis by different collaborators. Among the available compounds, structural diversity was also a selection criterion.

After determining their activity on proliferating parasites (IC_50_), the best compounds were tested in the Quiescent-stage Survival Assay (QSA) [28] demonstrating the efficiency of some epidrugs against artemisinin-resistant parasites at the quiescent state.

## 2. Materials and Methods

### 2.1. Chemical Compounds

Thirty-two compounds synthetized to target epigenetic pathways were studied (Figure 1). BIX-01294, apicidin, chaetocin, SGI-1027 and hydralazine were obtained from Sigma-Aldrich (St. Louis, MO, USA). TM2-115 was first synthetized at Imperial College London (M. Fuchter’s laboratory), United Kingdom and then obtained from Aobious (Gloucester, MA, USA). Trichostatin A, JIB-04, MG149 and C646 were obtained from Adooq Biosciences (Irvine, CA, USA). CB3717 was kindly provided by the Developmental Therapeutics Program, Division of Cancer Treatment and Diagnosis, National Cancer Institute (Frederick, MD, USA). SAHA, JAHA, PCI 34051, TC-H 106, MC 1568, tubastatin A, (+)-JQ-1, (+)-JD-1, SHH-878-1, AKE-040 [34] were from John Spencer’s laboratory and MZ1 was obtained from Biotechne, Tocris (Avonmouth, UK); https://www.tocris.com/products/mz-1_6154, accessed on 1 June 2021). Flv69 [35], Flv880 [36], MLo1302, MLo1401, MLo1406, MLo1502, MLo1507, MLo1508, MLo1509 [37] and NFlav2018 synthesized following our previously described procedures. Different batches of apicidin, BIX-01294 and TM2-115 were used during this work.

Artemisinin (ART) and atovaquone (ATQ) were purchased from TCI. Dihydroartemisinin (DHA) was provided by the WorldWide Antimalarial Resistance Network reference standards programme (WWARN, Bangkok, Thailand).

All compounds were prepared as stock solutions in 100% DMSO (Sigma-Aldrich).

### 2.2. Parasite Culture

F32-ART, an artemisinin-resistant strain, and F32-TEM, its artemisinin-sensitive twin line, were obtained as previously reported [9,28]. They differ by eight mutations in seven genes (PF3D7_0110400, PF3D7_1343700, PF3D7_0213400, PF3D7_1115700, PF3D7_1302100, PF3D7_1459600, PF3D7_1464500) among them the M476I mutation on the *kelch13* gene. F32-ART and F32-TEM strains have RSA^0–3 h^ values up to 12% and less than 0.1%, respectively [9,28]. The F32-ART strain, which has been submitted to more than 100 artemisinin pressure cycles, provides a relevant model of artemisinin resistance obtained after long-term drug pressure similar to resistant parasite isolates from the field. Both strains were cultured, in the same conditions, according to the Trager and Jensen protocol with modifications [28,38]. Briefly, parasites were cultivated in RPMI-1640 medium (Biowest) at 2% hematocrit in human red blood cells and 5% human serum (EFS, French Blood Bank) under 5% CO_2_ in a humid atmosphere at 37 °C. Parasites studied were routinely synchronized at the ring stage, by treatments with 5% D-sorbitol solutions, in parallel for both strains in order to have homogenous parasite populations.

### 2.3. Standard Chemosensitivity Assay

The antimalarial activities of the compounds were evaluated on both strains. Artemisinin (ART; TCI Europe N.V.), dihydroartemisinin (DHA; WWARN, Bangkok, Thailand) and atovaquone (ATQ, TCI) were used as reference compounds. Antimalarial activities were determined with the SYBR Green I method. Each drug concentration was tested in triplicate. The parasites were incubated with the drugs for 48 h. Plates were read on a BioTek FLX800 Microplate Fluorescence Reader (Bio-Tek instruments, Winooski, VT, USA) (λexcitation = 485 nm, λemission = 528 nm). Control parasite culture (i.e., RPMI with 0.5% DMSO) was referred to as 100% growth. IC_50_ and IC_90_ values were determined using GraphPad Prism version 7.05 (GraphPad Software, San Diego, CA, USA). Results were the mean of 3 independent experiments. When it is relevant, statistical significance between results obtained with both parasite strains was determined by a Mann–Whitney test using GraphPad Prism version 7.05.

### 2.4. Cytotoxicity Evaluation

The cytotoxicity of all newly synthesized compounds was evaluated on the Vero cell lines (isolated from monkey kidney cells, gift from the Faculty of Pharmacy, Toulouse, France) by MTT assay according to Mosmann [39] with slight modifications. Briefly, cells in MEM medium supplemented with 10% fetal calf serum, 2 mM L-glutamine, antibiotics (100 U/mL penicillin and 100 µg/mL streptomycin), and NEAA 1X, were seeded in 96-well plates. After a 24-h incubation at 37 °C and 5% CO_2_ in a humidified atmosphere, compounds were added with concentrations ranging from 5 nM to 50 μM in duplicate. Artemisinin, used as the antiplasmodial control molecule, was tested up to 100 µM. For compounds for which cytotoxicity was already published, literature data were directly reported in Table 1. The security index was calculated as the ratio of cytotoxicity/antiplasmodial activity.

### 2.5. Quiescent-Stage Survival Assay (QSA)

The sensitivity of the ART-resistant parasites F32-ART at the quiescent stage towards the different molecules was determined using the QSA (Quiescent-stage Survival Assay), as previously described [28]. It is important to note that we previously demonstrated that QSA results obtained with the ART-resistant laboratory strain F32-ART were similar to those obtained with ART-resistant field isolates [28] and whatever the culture conditions (5% human serum, 5% CO_2_, 95% atmospheric air vs. 0.55% albumax, 5% human serum, 5% O_2_, 5% CO_2_ and 90% N_2_).

Epidrugs were tested at their IC_90_ values. In the present study, quiescence was induced using 3 µM of ART or 700 nM of DHA [28]. Thus, D-sorbitol synchronized ring-stage parasites at 3% parasitemia and 2% hematocrit (5 mL of culture per well in 6-well plates) were first exposed to 6 h of ART or DHA to induce quiescence and then to 48 h of the combination “ART or DHA + compound to be tested”. The combinations “ART 6 h/ART 48 h” or “DHA 6 h/DHA 48 h” were used as controls. The compounds were evaluated, in parallel, on proliferating parasites, i.e., without any pre-treatment. After drug exposure, parasite cultures were washed with 40 mL of RPMI-1640 medium and cultured in drug-free conditions supplemented with 10% human serum in new wells. The parasitemia for each condition was then microscopically monitored every 2 days. Culture media were renewed twice a week and 25 µL of fresh red blood cells were added once a week. Obtained data were used to perform Kaplan–Meier survival analyses considering censored data. The outcome was defined as the day when parasite cultures reached the initial parasitemia (3%) and data were considered as censored if no parasite recrudescence was observed during the 30-day experiment timeframe. When relevant, statistical significance between results obtained for the conditions “ART/ART” and “ART/ART + compound to be tested” or “DHA/DHA” and “DHA/DHA + compound to be tested” was determined by a Log Rank (Mantel Cox) test using GraphPad Prism version 7.05. For the best compounds targeting histones, in order to compare western blots data, modified QSAs were carried out by comparing “DHA 6 h/DHA 24 h” versus “DHA 6 h/DHA + compound to be tested 24 h”.

### 2.6. Determination of Abundance of Methylated and Acetylated Histone

Abundance of methylated and acetylated histone was determined in parasite cultures in different conditions: “not-treated”, “DHA 24 h”, “DHA 6 h/DHA 24 h” and “DHA 6 h/DHA + compound to be tested 24 h”. At the end of the treatments, parasite cultures were washed in RPMI-1640 and then twice in PBS 1× before red blood cell lysis with 0.075% saponin (Sigma-Aldrich) in PBS. Parasite ghosts were washed 3 times in PBS before incubating the ghosts with E1A lysis buffer (50 mM Hepes pH 7.5; 250 mM NaCl; 5 mM EDTA; 0.1% Triton X-100, 1 mM DTT, 0.2 mM PMSF supplemented with complete Mini EDTA-free protease inhibitor cocktail (Roche^®^, Basel, Switzerland)) for 15 min. Ghosts were then sonicated for 3 × 15s ON/OFF cycles to shear parasite membranes. Debris were pelleted by centrifuging for 15 min at 13,800× *g* and 4 °C, and supernatants (containing parasitic proteins) were then collected. Protein concentrations were determined using BCA assay (Bio-Rad, Hercules, CA, USA) according to the manufacturer’s instructions. For each sample, 10 µg of proteins were mixed with 4× sample buffer (10 mL 50% Glycerol, 5 mL 0.5 M Tris HCl pH 6.8, 4 mL 20% SDS, 0.02% Bromophenol blue for a total solution volume of 20 mL) and 100 mM DTT and then separated on 15% SDS-PAGE gels in Laemmli running buffer. Gels were then wet-transferred O/N at 20 V onto PVDF membranes. Membranes were blocked in TBST for 1 h at room temperature. Bottom parts were then probed with histone antibodies while upper parts were probed with PFALDOLASE antibody (Abcam, ab38905, Cambridge, UK) at 1:20,000 dilution. The following primary antibodies were used: anti-H3K4me1 (Abcam, ab8895); anti-H3K4me2 (Abcam, ab35356); anti-H3K9me2 (Abcam, ab1220); anti-H3K9me3 (Millipore, 07-442, Burlington, MA, USA); anti-tetra-acetyl H4 (Millipore, 06-866); anti-H4K8Ac (Millipore, 07-328); anti-H4K20me3 (Abcam, ab9053); anti-H3K4me3 (Diagenode) and anti-H3K9Ac (Millipore, 07-352) at 1:10,000. The following secondary antibodies were used: anti-rabbit IgG-Peroxidase produced in goat (Sigma-Aldrich) or anti-mouse IgG-Peroxidase (Sigma-Aldrich); at 1:20,000 dilutions. Blots were developed using ECL Prime Western Blotting (GE Healthcare, Chicago, IL, USA) and read on a Chemidoc touch imaging software. Densitometry analysis was performed by using ImageLab software from Bio-Rad. To determine if variations in histone abundances were statistically significant, immunoblotting experiments were performed on 3 biological replicates and each run was performed twice.

## 3. Results and discussion

### 3.1. Antiplasmodial Activity of Some Epidrugs

The 32 epidrugs selected for this work were chosen for the variety of their respective targets (HDACs, HATs, HMTs, HDMs, BRDs, DNMTs) and also of their different chemical structures (i.e., cyclic tetrapeptides, hydroxamates, L-cysteine derivatives, quinazoline derivatives…) (Figure 1). Epidrugs were first screened in the standard chemosensitivity assay to determine their activity on both ART-resistant and ART-sensitive *P. falciparum* strains, respectively F32-ART and F32-TEM. Twenty-two compounds had IC_50_ values above 1 µM, while ten molecules (SAHA, JAHA, trichostatin A, apicidin, BIX-01294, TM2-115, chaetocin, CB3717, JIB-04 and SGI-1027) had antiplasmodial activity with IC_50_ values ranging from 29 nM to 1 µM (Table 1). For all molecules already known to have antiplasmodial activity, IC_50_ values obtained here were consistent with those previously published with other strains [43,48,56,57]. Interestingly, no significant differences of IC_50_ values were noted between ART-resistant and ART-sensitive strains, suggesting that ART-resistance does not seem to impair epidrug efficacy.

### 3.2. Specific Antiplasmodial Activity of Certain Epidrugs

The cytotoxicity evaluation of the 32 compounds showed CC_50_ values (concentration reducing the proliferation of cells by 50%) from 0.125 µM to >50 µM. This large disparity of values can be explained by the fact that several inhibitors were initially designed not to target parasites but human cancer cells leading to strong activity on mammalian cells. The selectivity indexes (SI) calculated by the ratio of cytotoxicity/antiplasmodial activity ranged from 0.01 to >790. Until now, no PROTAC has been developed for protozoan parasites [58], so the good SI (SI: >30) of MZ1 is promising. However, the weak antiplasmodial activity of MZ1 (IC_50_: 1.3–1.7 µM) suggested precluding this molecule from further assays. Among the ten compounds with antiplasmodial activity ≤1 µM, only chaetocin was not selective. The other ones have SIs ranging from 5 to >790 and the compounds with the best SIs were TM2-115 (SI: 45), BIX-01294 (SI: 110), apicidin (SI: 280) and SGI-1027 (SI: >790). Except for the five compounds targeting bromodomains, these results show that, for each class of epigenetic effectors, one or several inhibitors are specifically active against *P. falciparum* in vitro confirming epigenetic modifiers as therapeutic antiplasmodial targets.

### 3.3. Some Epidrugs Are Active against ART-Resistant Parasites in a Quiescent State

The 10 compounds with IC_50_ values below or equal to the 1 µM threshold were tested in the QSA [28] to determine their activity against ART-resistant parasites maintained in a quiescent state during a 48-h exposure. Compounds were tested at their IC_90_ values of the concentration at which apicidin in a previous study showed a significant effect on epigenetic marks and gene expression [59]. Despite the potential risk of off-target activity, we used IC_90_ values for all epidrugs tested in the QSA to have comparable experimental conditions. As a reminder, activity against quiescent parasites is evidenced in the QSA by a significant delay in recrudescence time between the conditions “ART/ART” and “ART/ART + compound to be tested” (or “DHA/DHA” and “DHA/DHA + compound to be tested” when using DHA instead of ART to induce quiescence). The longer this delay, the more effective the compound is. However, to make sure this delay was meaningful and biologically relevant, we set a 6-day threshold to consider a compound to be active against quiescent parasites [28]. Atovaquone, used as a positive drug control in the QSA [28], showed delayed parasite recrudescence times ranging from 9 to 12 days.

**Figure 2:**
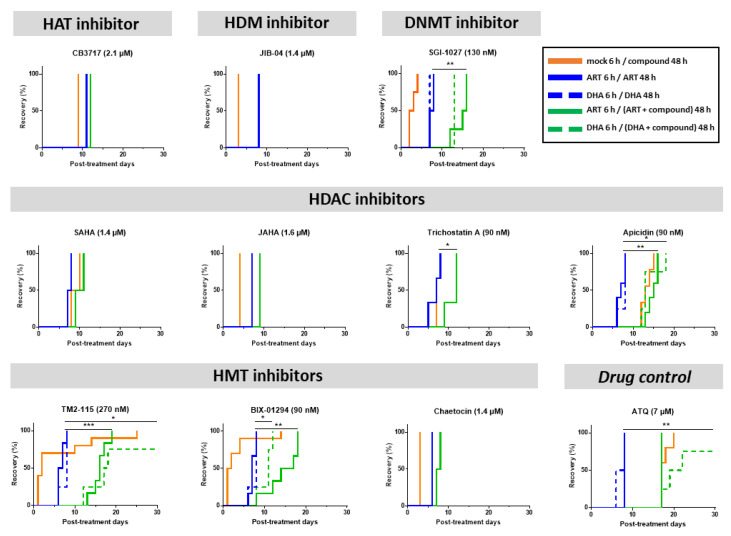
Evaluation of the activity of epidrugs towards F32-ART parasites in the Quiescent-stage Survival Assay (QSA) after a 48-h exposure. Kaplan–Meier survival curves showing recrudescence days after different drug treatments (ART is used at 3 µM, DHA at 700 nM and the molecules at their IC_90_ values). When appropriate, statistical significance was ascertained by using a log-rank (Mantel–Cox) test. * = *p*-value < 0.05; ** = *p*-value < 0.01; *** = *p*-value < 0.001. Solid lines correspond to ART treatment and dashed lines correspond to DHA treatment. Atovaquone (ATQ; targeting mitochondrial respiration) was used as antiplasmodial drug control active against quiescent parasites [28] and tested at 7 µM corresponding to its plasma peak concentration in patients. CB3717, JIB-04, SAHA, JAHA, trichosatin A and chaetocin were only tested in combination with ART. For JIB-04, the blue and green lines are superimposed.

Among the ten epidrugs tested in combination with ART, four (SGI-1027, apicidin, TM2-115 and BIX-01294) fulfilled these criteria in the QSA revealing their activity against ART-resistant parasites in a quiescent state (Figure 2). Indeed, the DNMT inhibitor SGI-1027 and the HDAC inhibitor apicidin delayed parasite recrudescence time by 8 days (difference between median recrudescence time of each condition) with *p*-values < 0.01. The two HMT inhibitors BIX-01294 and TM2-115 delayed parasite recrudescence time by 8.5 (with *p*-values < 0.01) and 9.5 days (with *p*-values < 0.001), respectively (Figure 2). When tested in combination with DHA instead of ART, similar results were obtained for TM2-115 and SGI-1027, with delays in recrudescence time ≥ 6 days, while apicidin and BIX-01294 showed a slightly shorter delay.

Therefore, among the 10 epidrugs tested against ART-resistant parasites at the quiescent state, SGI-1027, apicidin, TM2-115 and BIX-01294 have provided promising results with activities similar to atovaquone, known to target and eliminate the persister parasites in the QSA by targeting mitochondrial respiration still active during quiescence [14,24,60].

As QSA allows for the identification of compounds that are able to eliminate quiescent parasites and so is able to determine whether their targets are still active in this particular state, our results suggest that epigenetic effectors could still be functional in ART-resistant quiescent parasites.

### 3.4. Effects of Some Epidrugs on Histone Methylation and Acetylation in DHA-Treated Parasites

Epidrug activity against quiescent parasites suggests a role for epigenetic effectors in the survival of ART-resistant parasites upon artemisinin exposure. Therefore, the effect of (i) apicidin on histone acetylation, and (ii) BIX-01294 and TM2-115 on histone methylation was explored in the entire population of DHA-treated parasites (containing the quiescent ones), as the current method for sorting and recovering quiescent parasites does not allow us to obtain a sufficient quantity of material to do this analysis only on quiescent parasites by immunobloting. To collect enough material for immunoblotting, we performed the analysis after a 24-h exposure of DHA-treated parasites to the epidrug and in parallel evaluated the activity of the three epidrugs in a modified QSA, in which quiescent parasites were exposed to the drug to be tested for 24 instead of 48 h, as in our previous experiments (Figure 2).

**Figure 3:**
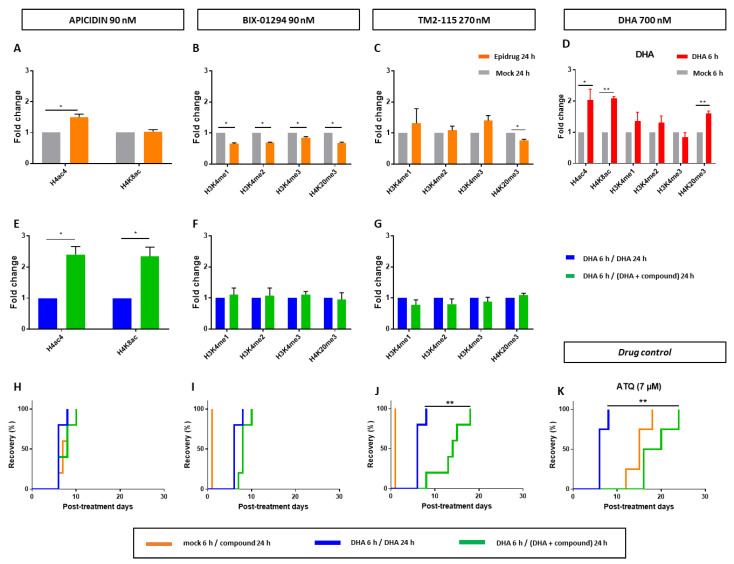
Evaluation of the activity of epidrugs (apicidin, BIX-01294 and TM2-115) towards F32-ART parasites. (**A**–**G**) Immunoblotting experiments on F32-ART parasites. Bar plots correspond to densitometry analyses normalized to PfALDOLASE levels. Densitometry values are from the analysis of western blots from 3 independent experiments and are the mean ± SEM normalized to the “DHA 6 h/DHA 24 h” condition for each treatment. Statistical significance was determined through a two-tailed paired *t*-test (* = *p*-value < 0.05). All the statistical analyses were performed by using GraphPad Prism software version 7.05 (GraphPad Software, San Diego, CA, USA). Full western blots are shown in the Supplementary Materials S1. (**H**–**K**) Evaluation of the activity of epidrugs towards F32-ART parasites in the Quiescent-stage Survival Assay (QSA) after a 24-h exposure. Kaplan–Meier survival curves show recrudescence days after different drug treatments (DHA is used at 700 nM and the molecules at their IC_90_ values). When appropriate, statistical significance was ascertained by using a log-rank (Mantel–Cox) test. * = *p*-value < 0.05; ** = *p*-value < 0.01. Atovaquone (ATQ; targeting mitochondrial respiration) was used as an antiplasmodial drug control active against quiescent parasites [28] and tested at 7 µM corresponding to its plasma peak concentration in patients.

Firstly, as control conditions, histone methylation and acetylation were quantified in proliferating parasites after treatment with the epidrug or with DHA alone (Figure 3A–D).

Apicidin, as a HDAC inhibitor, led to the increase of H4ac4 abundance in proliferating parasites (Figure 3A), as previously described [59], and also to the increase of both H4ac4 and H4K8ac in DHA-treated parasites (Figure 3E). Noteworthy, the first 6-h treatment with DHA alone already induced an increased level of these two histone marks (Figure 3D), meaning that apicidin amplified this response. However, 24-h exposure is not sufficient to affect quiescent parasite survival since no difference in recrudence time was observed in the modified QSA between the conditions “DHA 6 h/DHA 24 h” and “DHA 6 h/DHA + apicidin 24 h” (Figure 3H). The increase in acetylation might need to be sustained for a longer period of time, 48 h according to our results, in order to see any effect on parasite recrudescence.

BIX-01294, as a HMT inhibitor, led, in proliferative parasites, to a significantly decreased level of all studied methylation marks, while its structural analogue TM2-115 inhibited H4K20 methylation but not that of H3K4 (Figure 3B,C), contrary to what was previously described [48]. This difference could be due to variations in treatment protocols or in the parasite strains studied. For example, in the study reported by Malmquist et al. [48], TM2-115 was used at 1 µM, while it was used here at a concentration of 270 nM, which corresponds to its IC_90_ value. DHA-treatment alone caused an increase in H4K20 methylation (Figure 3D). In DHA-treated parasites, adding BIX-01294 or TM2-115 treatments did not induce a quantifiable change in the levels of any studied methylation marks (Figure 3F,G). Despite that, in the same experimental conditions, TM2-115 clearly impaired quiescent parasite survival already at 24 h according to QSA assays (Figure 3J).

## 4. Conclusions

Out of the 32 epidrugs tested, the four compounds SGI-1027, apicidin, TM2-115 and BIX-01294 were found to be active on parasites in proliferation but also when they were in a state of quiescence induced and maintained by DHA. Altogether, our results reinforce the potential of epidrugs as antiplasmodial drugs with good activity and selectivity toward *Plasmodium falciparum*, as starting points for new possible drug candidates. In the current context of ART-resistance emergence in Africa, the ability of epidrugs targeting different epigenetic mechanisms to impair recrudescence of ART-resistant and quiescent parasites is of particular interest. This underlines histone methylation, DNA methylation and histone acetylation as promising targets to kill ART-resistant parasites, whatever their metabolic state, which is mandatory for malaria eradication.

## Figures and Tables

**Table 1:** Susceptibility of *Plasmodium falciparum* F32-ART and F32-TEM lineages to different inhibitors involved in the epigenetic regulation of gene expression.

	Drug	Antiplasmodial Activity: IC_50_ (nM)		*p*-Value ^1^ (F32-ART vs. F32-TEM)	Cytotoxicity: CC_50_ (µM) (Cell Type)	Selectivity Index ^2^ CC_50/_IC_50_
		**F32-ART**	**F32-TEM**			
HDAC inhibitors	Trichostatin A	29 ± 12	30 ± 3	0.8	0.2 (NFF) [40] ^3^	7
	Apicidin	36 ± 9	36 ± 5	>0.99	10 (Jurkat) [41] ^3^	280
	JAHA	460 ± 30	690 ± 80	0.1	2.4 (MCF7 cancer) [42] ^3^	5
	SAHA	540 ± 55	390 ± 10	0.1	5.5 (NFF) [43] ^3^	10
	Tubastatin A	7 × 10^3 ^*	8 × 10^3 ^*		>10 (661W) [44] ^3^	>1.4
	PCI 34051	8 × 10^3 ^*	9 × 10^3 ^*		>20 (MCF7) [45] ^3^	>2.5
	TC-H 106	>10 × 10^3^ *	>10 × 10^3^ *		6.3 (GM15850) [46] ^3^	<0.6
	MC 1568	>10 × 10^3^ *	>10 × 10^3^ *		10 (monocytes) [47] ^3^	<1
HMT inhibitors	BI × -01294	56 ± 8	55 ± 14	>0.99	6.1 (HFF) [48] ^3^	110
	TM2-115	128 ± 30	104 ± 32	0.4	5.7 (HFF) [48] ^3^	45
	Chaetocin	640 ± 160	730 ± 90	>0.99	0.13 (HL-60) [49] ^3^	0.2
HAT inhibitors	CB3717	1 × 10^3^ ± 0.16 × 10^3^	1 × 10^3^ ± 0.11 × 10^3^	>0.99	>20 (NIH3T3) [50] ^3^	>20
	MG149	>10 × 10^3^	>10 × 10^3^		51 (NOMO1) [51] ^3^	<1.7
	C646	>10 × 10^3^	>10 × 10^3^		Between 10 and 20 (WM983A) [52] ^3^	<1
HDM inhibitor	JIB-04	560 ± 80	940 ± 30	0.1	>10 (human mesenchymal stem) [53] ^3^	>18
DNMT inhibitors	SGI-1027	63 ± 13	54 ±22	0.7	>50 (H4IIErat) [54] ^3^	>790
	MLo1302	7 × 10^3^ ± 2 × 10^3^	8 × 10^3^ *		6.5 * (Vero)	0.9
	Hydralazine	>10 × 10^3^	>10 × 10^3^	0.4	40 (HeLa) [55] ^3^	<4
	Flv69	>10 × 10^3^ *	>10 × 10^3^ *		>50 * (Vero)	
	Flv880	>10 × 10^3^ *	>10 × 10^3^ *		>50 * (Vero)	
	MLo1401	>10 × 10^3^ *	>10 × 10^3^ *		20 * (Vero)	<2
	MLo1406	>10 × 10^3^ *	>10 × 10^3^ *		>50 * (Vero)	
	MLo1502	>10 × 10^3^	>10 × 10^3^ *		>50 * (Vero)	
	MLo1507	>10 × 10^3^ *	>10 × 10^3^ *		40 * (Vero)	<4
	MLo1508	>10 × 10^3^ *	>10 × 10^3^ *		36 * (Vero)	<3.6
	MLo1509	>10 × 10^3^ *	>10 × 10^3^ *		>50 (Vero)	
	NFlav2018	>10 × 10^3^ *	>10 × 10^3^ *		>50 * (Vero)	
Bromodomain inhibitors	(+)-JD-1	6 × 10^3^ *	4.5 × 10^3^ *		0.6 * (Vero)	0.1
	SHH-878-1	10 × 10^3^ *	10 × 10^3^ *		3 * (Vero)	0.3
	(+)-JQ-1	>10 × 10^3^ *	>10 × 10^3^ *		0.2 * (Vero)	<0.02
	AKE-040	>10 × 10^3^ *	>10 × 10^3^ *		24 * (Vero)	<2.4
PROTAC BRD4 degrader	MZ1	1.7 × 10^3^ ± 0.3 × 10^3^	1.3 × 10^3^ *		>50 * (Vero)	>30
Control drug	artemisinin	14 ± 3	13±3	0.5	>100 (Vero)	>7500

IC_50_: 50% inhibitory concentration against *Plasmodium falciparum.* CC_50_: 50% inhibitory concentration against mammalian cells. All data correspond to the mean of three independent experiments ± SEM, except those noted * corresponding to the mean of two independent experiments. ^1^ Mann–Whitney test. A *p* value < 0.05 was considered statistically significant. ^2^ The selectivity index was calculated as the ratio cytotoxicity/antiplasmodial activity on F32-ART. ^3^ Data from literature.

## Data Availability

Data are contained within the article and in Supplementary Materials.

## Supplementary Materials

The following supporting information can be downloaded at: https://www.mdpi.com/article/10.3390/pharmaceutics15102440/s1, File S1: Immunoblotting.
